# Medical Education

**Published:** 1884-11

**Authors:** William S. Tremaine

**Affiliations:** Professor of the Principles and Practice of Surgery


### BUFFALO

### Medical and Surgical Journal.

VOL. XXIV. NOVEMBER, 1884. No. 4.

### Original Communications.

### MEDICAL EDUCATION.

AN ADDRESS DELIVERED BEFORE THE STUDENTS OF NIAGARA MEDICAL COLLEGE, AT THE OPENING OF THE SESSION OF 1884–5.

BY WILLIAM S. TREMAINE, M. D.,
Professor of the Principles and Practice of Surgery.

"Life is short and the art long, the occasion fleeting, experience fallacious and judgment difficult."

These words were uttered over two thousand years ago by the father of medicine, the contemporary of Socrates and Plato, the wise, the learned physician of Cos, the immortal Hippocrates, and they are as true to-day as when, it may be, perchance, on some autumn afternoon in the academic groves of that island in the Grecian Archipelago, they were addressed to his pupils, together with others of his wonderful aphorisms, which have come down to us for our instruction and guidance.

In the march of civilization there seem to be moments when, after a long halt, the herald sounds his trumpet, and forthwith a leader appears, an intellectual champion, a genius, and this not in one, but in every department of human knowledge. He heads the column, and the march is resumed. Such a leader in the golden age of Grecian history was Hippocrates, the friend and contemporary of Plato and Socrates. Founded in the Socratic philosophy, inspired by the genius of Hippocrates, medicine continued to advance by the double pathway of observation and reason.

From Greece the art was carried to Rome, and the next great teacher who arrests the attention of the student of the history of medicine is Celsus, who was followed by Galen, both of whom resided in the Imperial City. After this epoch came a period of intellectual darkness. Another halt, until Bacon, with his inductive philosophy, revived the Hippocratic reasoning. But so great was the impulse given our art by the father of medicine that some progress continued to be made.

A knowledge of the art of surgery was obtained by the Arabs, who had conquered Alexandria, then the chief seat of learning. There were, at that time, three sects, the Empirics, the Rationalists and the Methodists. Of these the Rationalists were by far the most enlightened, and to them belonged the great Arabian physicians, Rhazes and Albucasis, together with the distinguished Greek surgeon, Paulus, of Equieta. In his hands, indeed, ancient surgery reached its climacteric; in his works many operations and diseases are described with a mental grasp and thoroughness scarcely exceeded at the present day.

Without wearying you further with an attempt to trace the history of medicine in detail, I remark that the prominent thought that strikes one in studying its history is the diverse schools and methods that arose from *à priori* reasoning, a great teacher here and there formulating a theory of life and disease and attempting to found a philosophical system thereon; such, for instance, as the semi-metaphysical system of Galen, the microcosm of Paraceleus, the iatro-physical, the iatro-chemical school, the "excitability doctrine" of Brown, familiarly known as the "Brunnonian system," the wild vagaries of Hahnemann, whose teachings were so ridiculous and absurd as to excite much opposition on the part of the medical profession, so that he was driven to appeal to the public and flatter its self-love by making it an arbiter on questions of science and technical matters, obviously beyond its knowledge. He was thus enabled to pose as a martyr to professional prejudice. These conflicting schools were clearly the outcome of the attempt to construct a universal system of medicine, based upon abstract speculation.

In the early part of the nineteenth century what we may regard as the modern school was formed, characterized by the use of the methods of research which obtain in physical science and the avoidance of theories, the fundamental idea being to abandon *à priori* reasoning, and to study closely the structure and functions of living normal tissues and organs, and the deviation from the normal, producing those changes in structure and functions known as disease. The founder of this school was undoubtedly John Hunter, who, by his voluminous researches in the domain of anatomy and pathology, stimulated, greatly, the modern scientific method of research. It must be admitted, I think, that greater advance in general pathology has been made in the last half century than in all preceding time. To this largely contributed the discovery of Schwann of cells and the cellular pathology based thereon by Virchow. Thus, for instance, instead of formulating a theory of inflammation, the actual behavior of the tissues in this condition was studied by the aid of the microscope. As you progress in your studies, you will not have expounded to you this or that theory, but you will be shown the actual phenomena that take place in the tissues of the living body. To pursue this method of investigation in the domain of biology and pathology, you will plainly see that some acquaintance, at least, with the physical sciences is necessary. We, therefore, demand of him, who, in the present day, aspires to be an educated physician, not only a mind trained to think accurately by that process intended as a means of mental development, which is usually termed a "liberal education," but a technical knowledge of physics and chemistry is necessary, before you can properly begin the study of medicine. The object for which it is presumed you have matriculated at this university is the study of medicine. Have you ever stopped to consider what this means? A great thinker of modern times has said: "It were well to pause, sometimes, and translate our words into thought." Probably, if I were to put the question to most of you, individually, you would reply: "Study medicine, why, of course I want to know how to cure disease;" and while I admit the importance for yourselves and the public of the desirability of the cure of disease, I say, alas! that this *alone* should be considered the study of medicine; and this leads me to say that we must discriminate between the pursuit of medical science, and the practice of the healing art. The first embraces a knowledge of all the physical sciences that may possibly assist us in investigating physiological or pathological processes, the functions of the different organs and tissues of the body, the methods of growth and repair, the phenomena of reproduction and development, the natural history of disease, which, in itself alone, is encyclopædic in character. All these may and ought to be known, and yet, in the full possession of this knowledge, and from it, not a faint idea is suggested of how to cure diseases. That belongs, in the present state of our learning, chiefly to what is termed the *art* of medicine. You will thus see that I divide this study of medicine into the *science* and the *art*, the former embracing what I have indicated to you, the latter that rational empiricism derived from the study of the action of remedial agents upon man and the lower animals, in which great progress was made long before the days of modern scientific research; and while it is true that the efficacy of many remedies was well known from empirical methods some thousands of years long past, no real progress can be made without an accurate knowledge of science. As we become familiar with the laws governing the action of living organisms now being developed by this modern inductive method, the practice of the healing art becomes more accurate; and thus, while I lay great stress upon *science* as a necessity to true advance, I must warn you against neglect of the *art*. I fear we can never hope to reduce the *art* of medicine to a mathematical formula, in which disease and remedy will represent one side of the equation and health the other. We deal with an infinite variety of unknown quantities and indefinite variables, impracticable if not impossible to reduce to any fixed expression that can have a practical value. We investigate the *inscrutable*, and fight the *inevitable*.

The conditions of disease, or, more properly speaking, perverted normal action, are highly complex. We have to contend with age, sex, temperament and previous history, elements for which allowance can, to some extent, be made, but we have to deal with idiosyncracies, with a thousand and one social surroundings which may ever baffle our calculations and stultify our foregone conclusions. You may ask, what is the use, then, of science? By science we know and understand disease. It is diagnostic. By art we treat it. It is therapeutic. Let me impress upon you that medicine is a faculty to be acquired, not a lesson to be learned; to be acquired by long and patient observation of complex phenomena, not yet reduced to the hard and inelastic formulæ of exact science. You will see that the tendency of past times was to search for principles. As these were and are unattainable by hypotheses, loose and plausible theories were adopted in their place, hence the quackery and "isms" of the profession itself, which have now become the property of the public, and, sad to say, of a certain proportion of medical practitioners who set up claims to be considered physicians. It a curious fact that the fallacies of the medical profession of a past generation become the belief of the public in a succeeding one. You will find, for example, that the humoral pathology is the current belief of the *profanum vulgus* of our day. This is one reason of their lack of discrimination between the modern scientific physician and the charlatan, with his "paths" and "isms;" nor is it peculiar to our day and generation, for as far back as 1229, when the Emperor Frederick II. required that physicians should study philosophy three years, then medicine and surgery five years, and then practice for some time under an older physician, before being let loose on the public, there existed a class of medical practitioners whose type we are familiar with to-day. They practiced solely for money, were ignorant of the study of medicine, and wanting in scientific knowledge. They are yet occasionally to be found in the drawing-rooms of the new rich, and the super-refined, the difference being that in that day they were not styled doctors, that honorable title being reserved for those who had complied with the conditions laid down by the universities or seats of learning, the term "university" meaning then, as it should now, and does in other countries, an institution where learned men teach the higher branches of the arts and sciences, and should not be assumed by any joint-stock concern intended for the rapid manufacture of medical tradesmen at so much a head. Think you the distinguished novelist, Lever, had such a product in his mind's eye when he wrote, referring to the elevation of medical men to the peerage: "I own that I am anxious to see some share of the high honors of the State bestowed upon a class which, whether simply regarded as numbering among them some of the ablest thinkers and writers of the age, or conferring more gratuitous services on the world than any other rank and condition together, are less recognized than any other. Among all the trained and polished intellects you assemble in the Upper House you have not got that special form of mind that distinguishes the doctor. You have not got a man whose daily life and labor is passed between the deepest problems of science and the practical workings of the great truths he has been considering. In knowledge of his fellow-men he is without a rival. His study of temperament is a study of character, and this not in one class or condition, but in every walk and rank, from the palace to the hovel. He is the one man with whom no fallacy can succeed that assumes to say what men in this or that station might do or think, for he knows them all."

Gentlemen, for years I have been an advocate for higher medical education, for more thorough training for our work. However desirable it may have been in earlier days that men with only a limited knowledge of the healing art should be provided for the sparsely settled districts, that day has long since passed. There are no longer "backwoods" to be found. The conveniences produced by modern science have penetrated to every hamlet. Shall the doctor alone be ignorant?

You live in a privileged time. Medicine is now more than ever tending towards a positive science. Over a quarter of a century has elapsed since I occupied your position as a pupil, but a short time in the history of science, and yet I may fairly say that the amount to be learned has, at least, doubled in that time.

How, then, I say, is it possible that a student can, in two courses of four months, acquire sufficient knowledge to intelligently understand or treat disease or injury. He cannot do it, and as a matter of fact, it is never done. No one could learn the merely mechanical trade of a shoemaker in that time, far less obtain even a rudimentary knowledge of that science which virtually embraces *all* other sciences.

When I contrast the advantages you possess with those I and my fellow-students had in the days of our pupilage, I must say I envy you your high privileges. While I was yet an undergraduate, Virchow published his cellular pathology, the microscope was in its infancy, the ophthalmoscope, laryngoscope and clinical thermometer were not used. I remember hearing ovariotomy (that triumph of modern surgery, which, it is estimated, was added 40,000 years to human life), stigmatized as a barbarous and unjustifiable operation. Within my time has developed the splendid achievements of antiseptic surgery, whereby almost any operation is rendered safe, and surgical mortality reduced 25 or 30 per cent. Certainly, these are results to be proud of. To what are they due? Certainly not to the *charlatan* or the *pathist.* They have been brought about by the patient, self-denying efforts of men with well-trained minds, imbued with the scientific spirit of search for *truth.* Will you profit by their labors without fitting yourselves by proper training to add your tribute, albeit, but a single stone, for the Temple of Science, in whose Holy of Holies is enshrined the mystery of life! While your privileges are great, your responsibilities are increased in proportion.

"The laboratory is the forecourt of the Temple of Philosophy; and whoso has not offered sacrifices, and undergone purification there, has little chance of admission into the sanctuary." So wrote one of the masters in science, Thos. Huxley. See to it that *your* garments are white.

The medical man must ever be a *student* for life. The first lesson, then, to learn, is *to study, how to study* and *what* to study. Early acquire the habit of thoroughness and method. Realize that the end and aim of your work is to acquire knowledge and intelligently apply it, and not simply to answer questions and pass examination. Learn to examine yourselves, that you gain knowledge understandingly. "Apply thine heart unto instruction, and thine ears to the words of knowledge," and remember, above all, that "He that getteth wisdom loveth his own soul, and he that keepeth understanding shall find good." Do not attempt too much in the beginning, bearing in mind that one subject thoroughly mastered will be of much greater ultimate advantage than a dozen superficially skimmed over. Question yourselves, or each other, as to the real progress in your daily work. To those of you who are beginning study, the three branches of anatomy, physiology and chemistry will sufficiently occupy your time. There is an erroneous impression that anatomy is only of use to the surgeon. It is of equal, if not greater, importance to the physician. You must then *know* your anatomy, and be able to answer any question, as Huxley said, as you would that of the arrangements of your own apartments. Avoid learning by rote, and endeavor, in studying the relations of any part, to understand, also, the purpose, it is to subserve in the animal economy. The same advice applies to physiology and chemistry. But I must not trespass on the domain of my colleagues. The incumbents of these several chairs will indicate to you, at the proper time and place, the manner in which you should pursue these special studies. Having a thorough knowledge of these, so to speak, *foundation subjects*, you will then be prepared to better appreciate the clinical lectures or bedside study of diseases; and each case, if systematically studied, will become a valuable and lasting lesson; and while I think it desirable that students should, early in their career, attend, to some extent, the clinics, I deprecate any attempt at systematic study of general pathology, practice of medicine and surgery, until well grounded in the *foregoing branches.* Above all, do not pursue your studies with a view to becoming specialists in conformity to the pernicious fashion of the day, whereby an organ prone to disease and suffering is singled out as if it were independent of the rest of the body and subjected to all kinds of torture and experiment—too often only an advertising trick. Methinks a future historian, possibly a contemporary of Macaulay's New Zealander, may indite an eloquent chapter on the relation between the decline and fall of nations and the evolution of the *gynecologist.*

In conclusion, I would add a few words in regard to the establishing of the medical department of Niagara University, which, to some of you, at least, will be your Alma Mater, and hence, during your future career, an object of special interest. In Europe the requirements for the degree of Doctor of Medicine are fixed by law, and, generally speaking, at a sufficiently high standard, accompanied by sufficient and enforced penalty against its unwarrantable assumption by unauthorized parties. It is conferred solely by chartered universities under government inspection and control and constituted, without a single exception, of what is known as regular medicine, ignoring, entirely, so-called "schools," or what are popularly understood as different systems of practice. A diploma given by one of these bodies means just what it says—that the possessor is well instructed in the science and art of medicine. It is the "Hall mark," indicating the quality of the article. It is to be regretted that the same is not true of that title in our country. Hosts of colleges swarm all over the land, issuing diplomas whose value bears the same relation to the genuine article that the spurious coin of the counterfeiter bears to that of the realm or republic respectively. Every "pathy" and medical heresy has its college or university (save the mark), often deluding the untaught public into the belief that their wares are genuine. Fancy a university designated by the prefix of a "path," the catholicity of science conditioned and bounded by an "exclusive dogma"! Perish the idea! Science can have no schools in this sense. It is possible that there are those who mistake the paths of science for "*paths*" of pseudo-science. Do not misunderstand me. I quarrel with no man because of his opinion or belief on unsettled matters, or his theories, provided he is well versed in those sciences which, by the common consent of the educated world, go to make up the learned physician, and is an earnest seeker after scientific truth for truth's own sake, but I do protest against the assumption of *trade-marks* in science. I trust that each of you will strive for that completeness of knowledge which is embraced in the simple title of a learned physician.

The profession in general is entirely awake to the deplorable looseness of our medical education. The public, too, are partially alive to the situation. In this country, where it seems impossible, or, at all events, impracticable to accomplish reform by legislation, the remedy must come from the profession itself. The time is ripe when it should lay its strong hand on the colleges and say: "We will not admit your graduates to our fellowship unless you demonstrate to us in some positive manner that they are worthy of recognition by being thoroughly trained in our science and art, which can only be done by taking sufficient time to pursue the numerous and varied studies that now constitute a regular medical education."

If the profession is in earnest in its desire for reform, it can best demonstrate it by supporting and encouraging teaching bodies which endeavor to meet these requirements. The Niagara University is one in fact as well as in name. Its trustees, belonging to an organization whose belief in thorough training for work is well known, have determined to make such an effort, and the day will come when its graduates will be able to point proudly to their diplomas as having a *real* and not fictitious value. May the true spirit of scientific inquiry so pervade your minds, and direct your studies, that wherever you go, or whatever tests you may be subjected to, you will always be living examples of the fulfillment of this prediction, and bear honorable testimony to the faithful teaching of your young but vigorous Alma Mater; for, as Lord Bacon says: "I hold every man a debtor to his profession, from which, as men, of course, do seek to receive countenance and profit, so ought they, of duty, to endeavor, themselves, by way of amends, to be a help and ornament thereto."

